# Detection of Pathogenic *Leptospira* DNA in Cervicovaginal Mucus of Mares With Reproductive Disorders in a Brazilian Herd

**DOI:** 10.1111/rda.70125

**Published:** 2025-09-24

**Authors:** Daiany Motta, Juliana Pedrosa, Walter Lilenbaum

**Affiliations:** ^1^ Laboratory of Veterinary Bacteriology, Biomedical Institute Federal Fluminense University Rio de Janeiro Brazil

**Keywords:** leptospirosis, mares, PCR, reproductive disorders

## Abstract

Leptospirosis is a zoonotic disease caused by pathogenic bacteria of the genus *Leptospira*. A lesser‐known form, equine genital leptospirosis (EGL), has been identified as a chronic and often silent infection involving the colonisation of the mare's genital tract. Despite its potential impact, EGL remains underdiagnosed and poorly understood, particularly in its association with reproductive inefficiency. This study showed the presence of *Leptospira* spp. DNA by *lip*L32‐PCR in the genital tract of mares with a history of reproductive disturbances. Cervicovaginal mucus samples were collected from 120 adult mares exhibiting recent reproductive problems. Results showed that 30 (25%) of the mares tested positive for *Leptospira* DNA. Among these 30 positive cases, 23.3% had experienced abortions, 3.3% had stillbirths, 53.3% showed placental alterations, and 36.6% were subfertile. These findings suggest a possible association between EGL and reproductive disorders in mares. The high detection rate of *Leptospira* DNA in genital samples reinforces the need for increased awareness and improved diagnostic efforts.

## 1 Introduction

Leptospirosis is an important zoonosis caused by pathogenic bacteria of the genus *Leptospira*, and affects humans, domestic, and wild animals (Karpagam and Ganesh 2020). Equine leptospirosis is frequently reported as ocular, hepatorenal, and pulmonary syndromes (Divers et al. 2019; Ramsay et al. 2024). However, the clinical manifestations in the reproductive tract are still poorly understood, particularly the chronic and silent genital infection, which is consequently underdiagnosed. Recently, a syndrome called equine genital leptospirosis (EGL) has been described as a silent and chronic disease characterised by the colonisation of leptospires in the genital tract of mares (Di Azevedo and Lilenbaum 2022). EGL leads to reproductive disorders such as low embryo retrieval rate, early embryonic loss, placentitis, fetal death, miscarriage and subfertility (Aymée, Dantas, et al. 2024; Malalana 2019).

Strains of the serogroup Australis, such as the Bratislava serovar, are known to be adapted to horses (Arent et al. 2015). It has been reported that infection with these strains causes a chronic and silent disease, in which the reproductive tract of mares is the main primary site of infection, responsible for subfertility and poor reproductive performance (Hamond et al. 2015). However, few studies have reported the association between the presence of leptospires in the genital tract and low reproductive efficiency, with EGL being still underdiagnosed and consequently neglected. Thus, the present study aimed to demonstrate the occurrence of genital infection by *Leptospira* sp. in mares with a clinical history of reproductive alterations.

## 2 Materials and Methods

### 2.1 Animals and Samples

A herd in the Brazilian Northeast had been showing pregnancy losses in the three previous breeding seasons. In the current season, the practitioner suspected of genital leptospirosis and selected 120 of the mares with reproductive problems for further investigation. Abortions and stillbirths represented 31.7% (38/120) of the losses, while placentitis was observed in 56.7% (68/120) of the mares. The other 14 mares (11.6%) presented placental retention and the birth of weak foals.

The CVM collection was performed with a cytological brush (Kolplast, Itupeva, SP, Brazil) attached to a cytologic device inside a vaginal speculum (Botupharma, Botucatu, SP, Brazil), and the brush was rubbed in the vaginal fornix to avoid urine contamination, as recommended for cows (Aymée, Reis, et al. 2024). The collected samples were immediately refrigerated and transported to the Veterinary Bacteriology Laboratory of the Fluminense Federal University, Brazil, for routine diagnosis of leptospirosis.

### 2.2 Reproductive Parameters

Besides history, reproductive examinations were performed by ultrasonography. The animals were classified as having placental alterations when varying degrees of placental edema were observed (increased combined thickness of the uterus and placenta CTUP for gestational age), placental abruption, thickening of the amniotic sac, and/or hyperechoic allantoic fluid (Aymée, Dantas, et al. 2024). Reproductive disorders were classified as embryonic loss (< 40 days of gestation), abortion (40–300 days of gestation), and stillbirth (> 300 days of gestation). Subfertility was considered when more than three inseminations were necessary to become pregnant (Reed et al. 2018).

### 2.3 Molecular Analysis

DNA extraction from CVM samples was performed with the DNeasy Blood and Tissue Kit (Qiagen, California, USA) following the manufacturer's protocols. PCR reactions were performed as described (Aymée, Reis, et al. 2024). Briefly, PCR targeted the *lipL*32 gene, reported to be present in pathogenic leptospires (Stoddard et al. 2009), and was conducted using primers LipL32_45F (5′AAG CAT TAC TTG CGC TGG TG 3′) and LipL32_286R (5′TTT CAG CCA GAA CTC CGA TT 3′), amplifying a 242‐bp fragment. In the thermal cycler, the reaction included one cycle of initial denaturation at 94°C for 2 min, followed by 40 cycles of denaturation at 94°C for 30 s, annealing the primers to 56°C for 30 s, and 1‐min extension at 72°C, followed by a final extension cycle at 72°C for 5 min. Ultrapure water and DNA from *L. interrogans* serovar Copenhageni (Fiocruz L1‐130) were used as negative and positive controls of the reaction, respectively. After amplification, PCR‐*lipL*32 products were subjected to 2% agarose gel electrophoresis and visualised under UV light after staining with red gel.

## 3 Results

This is a descriptive case series. Of the 120 mares presenting reproductive problems, 25% (30/120) were positive for *lip*L32‐PCR at CVM, confirming the presence of leptospiral DNA in the genital tract of the animals. Besides the reproductive problems, none of the animals showed clinical signs of acute systemic leptospirosis. Among the 30 CVM‐PCR positive mares, seven (23.3%) had abortion episodes, one (3.3%) had stillbirth, 16 (53.3%) had placental alterations, and 11 (36.6%) had subfertility (Table 1).

**TABLE 1:** Reproductive problems in 30 positive mares at CVM‐PCR for Leptospiral DNA.

Mare	PCR CVM	Reproductive changes
1	Positive	Placental alterations
2	Positive	Placental alterations
3	Positive	Stillborn and placental alterations
4	Positive	Placental alterations
5	Positive	Placental alterations
6	Positive	Abortion
7	Positive	Placental alterations
8	Positive	Placental alterations
9	Positive	Placental alterations
10	Positive	Placental alterations
11	Positive	Placental alterations
12	Positive	Placental alterations
13	Positive	Placental alterations
14	Positive	Placental alterations
15	Positive	Placental alterations
16	Positive	Abortion
17	Positive	Placental alterations
18	Positive	Placental alterations
19	Positive	Subfertility
20	Positive	Subfertility
21	Positive	Subfertility
22	Positive	Subfertility
23	Positive	Subfertility
24	Positive	Subfertility
25	Positive	Abortion
26	Positive	Subfertility
27	Positive	Abortion and subfertility
28	Positive	Abortion and subfertility
29	Positive	Abortion and subfertility
30	Positive	Abortion and subfertility

## 4 Discussion

In the present study, 30/120 (25%) of the CVM samples were *lip*L32‐PCR‐positive, an expressive number considering that none of the mares presented any other symptoms suggestive of leptospirosis, such as ocular, pulmonary, or systemic alterations. Equine Genital Leptospirosis (EGL) is a recently described syndrome and requires several additional studies to be better understood (Di Azevedo and Lilenbaum 2022). In parallel to Bovine Genital Leptospirosis, a similar syndrome in cows that has been receiving a lot more attention, EGL is characterised by a chronic and silent infection of genital organs, independent of kidney colonisation and manifested not by systemic symptoms, but exclusively by reproductive problems. In the present study, we could reinforce its occurrence in naturally infected mares with poor reproductive performance.

Although many strains are frequently reported as the most frequent agent of systemic acute leptospirosis in horses, with predominance of Icterohaemorrhagiae under tropical conditions, it has been hypothesised that adapted strains such as Australis and Bratislava (from the same serogroup) lead to the chronic colonisation of genital organs and consequent reproductive problems (Hamond et al. 2024; Browne et al. 2022).

Since colonisation occurs in the genital organs, mainly the uterus, urine or blood is not the best samples for the diagnosis. Instead, genital samples such as CVM or uterine biopsy are the most suitable. In the present study, we could successfully diagnose those mares as *Leptospira*‐infected using CVM. In cows, recent studies (Aymée, Reis, et al. 2024; Di Azevedo et al. 2020) suggest that MVC adequately reflects the health of the uterine environment, in addition to being easily collected under field conditions. Therefore, we believe that the MCV is a good sample for evaluating reproductive disorders also in mares.

Reproductive problems without other visible systemic symptoms are the main characteristic of EGL. In this study, we could register abortions, placental alterations, subfertility, and stillbirths. None of the animals in the study showed systemic signs of classic equine leptospirosis, such as uveitis and/or hepatorenal alterations. In this context, if EGL had not been suspected, those animals would not have been tested (using CVM) and would have remained without a diagnosis and certainly untreated. Since it was a study conducted on samples sent for diagnosis, the present study presents some limitations that are not commonly seen in planned studies. For example, we understand that a control negative group could be useful, and testing animals from different herds would also add value to the quality of the information. In addition, it is essential to consider that the absence of a differential diagnosis for other common causes of reproductive problems in mares, such as EHV‐1 or bacterial/fungal endometritis (*Streptococcus zooepidemicus* or *E. coli* ), also represents a significant limitation in this study, since reproductive loss is a multifactorial problem. Despite this, due to the scarcity of studies assessing EGL, we understand that the occurrence of mares with reproductive loss showing positive CVM‐PCR results deserves to be reported. It may be of interest to veterinarians of equine reproduction and may alert the field practitioners. In this context, it may help veterinarians to have a different look at leptospirosis as a cause of reproductive failure in mares, even in the absence of any other visible symptoms, increasing the number of diagnosed herds.

In conclusion, this descriptive study demonstrated the presence of pathogenic leptospiral DNA in genital samples from mares with a history of reproductive disorders, reinforcing the role of EGL in equine reproduction. However, further studies focusing on the immunopathogenesis of *Leptospira* in the uterine environment, as well as the most common strains, are necessary for a better understanding of this disease.

## Author Contributions

All authors contributed to the study conception and design. Material preparation and analysis were performed by Daiany Motta. The manuscript was written by Daiany Motta, Juliana Pedrosa and Walter Lilenbaum. All authors commented on previous versions of the manuscript. All authors read and approved the final manuscript.

## Ethics Statement

The study was performed on samples sent for diagnosis in the Laboratory of Veterinary Bacteriology of Federal Fluminense University, Brazil, so previous authorisation of the Ethics Committee is unnecessary.

## Conflicts of Interest

The authors declare no conflicts of interest.

## Data Availability

Research data is not shared.
